# Melatonin: its possible role in the management of viral infections-a brief review

**DOI:** 10.1186/1824-7288-39-61

**Published:** 2013-10-03

**Authors:** Michela Silvestri, Giovanni A Rossi

**Affiliations:** 1 Pediatric Pulmonology and Allergy Unit, Istituto Giannina Gaslini, Genoa, Italy

**Keywords:** Antioxidant, Inflammation, Encephalitis viruses, Respiratory syncytial virus

## Abstract

Melatonin, a versatile molecule, is synthesized by the pineal gland but also by other organs, including gastrointestinal tract, retina, thymus, bone marrow, and by leukocytes. Besides playing an important role in various functions of the body, including sleep and circadian rhythm regulation, melatonin also shows immunoregulatory, free radical scavenger and antioxidant functions. Because of these latter characteristics melatonin has also been found to be effective in fighting viral infections in a variety of experimental animal and *in vitro* studies. These data suggest a possible therapeutic potential of melatonin in human virus-induced disorders.

## Introduction

**Figure 1:**
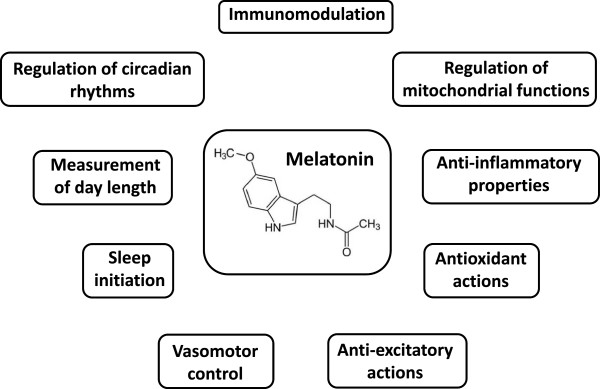
Main melatonin functions.

Melatonin (N-acetyl-5-methoxytryptamine) is the major neurohormone secreted by the pineal gland [1,2]. Initially, it was reported as a skin lightening agent in amphibians [3,4]. Further investigations showed that other functions of the molecule were the regulation and reset of circadian rhythms with involvement in the measurement of day length, an environmental variable used for seasonal timing of reproduction, metabolism and behavior in animal species [5-7] (Figure 1). Acting virtually in every cell in the organism, melatonin has been reported to possess numerous additional functions, being involved in sleep initiation, vasomotor control, anti-excitatory actions, regulation of mitochondrial functions [8]. Melatonin and its metabolites were found to have also important immunomodulatory and antioxidant properties owing to their direct and indirect antioxidant actions, i.e. by scavenging free radicals and by upregulating antioxidant pathways [9-12].

### Melatonin synthesis in the pineal gland and its metabolism

In mammals, melatonin is synthesized in the pineal gland with a rhythm regulated by an endogenous circadian clock, the most important factor regulating its metabolism being the light/dark cycle. Once formed, melatonin is immediately released into the cerebrospinal fluid and in the blood, with a half-life in serum varying between less than 30 min to 60 min, and then metabolized in the liver and in the kidney [13]. Melatonin exerts some of its actions via binding to specific receptors and intracellular targets [1,14]. There are two types of specific high affinity membrane-associated melatonin receptors, MT1 and MT2, able to trigger intracellular signaling by adenylate cyclase or G-proteins [1,14]. A third membrane-associated receptor, named MT3, has been described pharmacologically and characterized as the enzyme quinine reductase 2 [15]. This enzyme belongs to a group of reductases that participate in the protection against oxidative stress by preventing electron transfer reactions of quinines. High affinity nuclear receptors to melatonin have also been identified, belonging to the RZR/ROR nuclear hormone receptor family [16]. Additional binding to intracellular targets in the micromolar range, such as the enzymes hydroquinone [17] and calmodulin [18] have been reported, which mediate some of melatonin’s actions, including protection of the cytoskeletal organization from damage caused by free radicals [19]. In humans, melatonin is secreted rhythmically with low levels during the daylight hours and a peaks during darkness: in normal individuals, blood concentrations begin to rise during the evening, reaching maximum values between 02:00 and 04:00 am, and then return to baseline levels during the late morning [20]. Basal plasma melatonin levels may vary in several physiological conditions: as an example, they are elevated during pregnancy, reaching a maximum at term, and then return to “normal” levels soon after delivery [21].

### Modulation of immune-inflammatory processes and antioxidant activity

Besides being produced by the pineal gland, melatonin is also synthesized by many other organs like gastrointestinal tract, retina and leukocytes, both in the peripheral blood and in the bone marrow [1,22]. As an example, human lymphoid cells are an important physiological source of melatonin since resting and phytohemagglutinin-stimulated human lymphocytes synthesize and release large amounts of melatonin, with the melatonin concentration in the medium increasing up to five times the nocturnal physiological levels in human serum [23]. Melatonin produced by these non-endocrine organs is not regulated by circadian cycles but rather respond to other signals, exerting a paracrine or autocrine effect that superimpose on the neuroendocrine hormone response [23]. T-lymphocytes, natural killer (NK) cells, eosinophils, and mast cells possess melatonin receptors [22]. Melatonin has the capability to regulate leukocyte function and contributes to the control of inflammation in tissues acting as both an activator and inhibitor of the inflammatory and immune responses [1,2,24,25]. Melatonin administration increases the proliferative response of rat lymphocytes, increases the number of NK cells, stimulates the release of pro-inflammatory cytokines interleukin (IL)-1 and tumor necrosis factor (TNF)-α, enhances phagocytosis and modulates apoptosis [1]. On the contrary, in other experimental systems, melatonin inhibits translocation of nuclear factor-kappa B (NF-kB) to the nucleus, thereby reducing the upregulation of pro-inflammatory cytokines [26]. In addition, melatonin is able to prevent or reduce the inflammatory-derived activation of a variety of enzymes, including phospholipase A2, lipoxygenase, and cyclooxygenases [1].

Melatonin is also a powerful antioxidant since it has been reported to scavenge different types of free radicals *in vitro*, in body fluids and in cells [1,27]. Indeed, the activity and the expression of antioxidant enzymes such as superoxide dismutase, glutathione, catalase, glutathione peroxidase, and glutathione reductase have been shown to be increased by melatonin, supporting its indirect antioxidant action [1,27,28]. Further evidence of the antioxidant effect of melatonin is provided by its ability to reduce lipid peroxidation, a degradative phenomenon involved in the pathogenesis of many diseases [10]. In addition, melatonin can act on energy metabolism, stimulating mitochondrial biogenesis, increasing the efficiency of the electron transport chain in mitocondria, thereby limiting electron leakage and free radical generation [29,30]. Finally melatonin can increase mitochondrial glutathione levels, leading to protection against free oxygen species [31].

### Protective effect of melatonin in experimental infections due to encephalitis viruses

Because of its activity on the central nervous system (CNS), associated with its capability to regulate immune function and to act as powerful free-radical scavenger, melatonin was thought to be able to play a protecting role in infections induced by encephalitis viruses [32]. In this context, melatonin was shown: a) to prevent paralysis and death in mice infected with encephalomyocarditis virus, a highly pathogenic and aggressive virus that causes encephalitis, but also myocarditis, in rodents [32,33]; b) to reduce viremia and significantly postpone the onset of the disease and death in mice infected with the lethal Semliki Forest virus, a classic encephalitis arbovirus that invades the CNS and whose replication in the mouse brain eventually leads to death [34]; c) to attenuate noninvasive West Nile virus-induced disease, counteracting the immunodepressive effect of stress exposure, and to prevent the stress-related encephalitis and death of the infected mice [34]; d) to decrease the virus load in the brain and in serum of mice infected with Venezuelan equine encephalomyelitis virus, reducing mortality rates, delaying the onset of the disease and deferring the time to death [35]. All these studies suggest the concept that the protective mechanisms of melatonin against infections due to encephalitis viruses is probably due to a variety of functions, including the antioxidant activity and the ability to regulate immune functions inhibiting an excessive inflammatory response.

### Protective effect of melatonin in respiratory syncytial virus (RSV) infection

**Figure 2:**
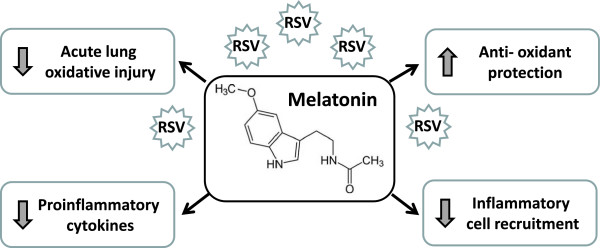
Melatonin in experimental Respiratory Syncytial Virus infection.

Results confirming the antioxidant activity of melatonin were obtained in studies performed on the RSV infection [36] (Figure 2). RSV is a common cause of bronchiolitis, a severe lower respiratory tract disease that infects nearly all infants by age three worldwide [36,37]. This disorder is characterized by an extensive damage to the bronchial epithelial cells and by massive infiltration and activation of inflammatory cells into the airways with production of reactive oxygen species [38]. Indeed, mice inoculated intranasally with RSV showed elevation of oxidative stress due to rises in nitric oxide (NO), hydroxyl radical (•OH) and malondialdehyde (MDA), associated with an opposite decreases in glutathione (GSH) and superoxide dismutases (SOD) activities. Pre-treatment of the animals with melatonin resulted in marked reduction of acute lung oxidative injury, with suppression of NO, •OH and MDA generation and restoration of GSH and SOD levels in the lung [36]. In RSV-infected mice, inhibition of oxidative stress was also associated with a reduced production of proinflammatory cytokines, such as TNF-α [36]. Although “physiologically controlled” inflammation is required for virus clearance, aberrant and exaggerated inflammatory reaction during RSV infection results in an extensive parenchymal damage with development of severe diseases, such as bronchiolitis and pneumonia [37]. TNF-α is a prooxidant cytokine able to amplify the host inflammatory response to respiratory viral infections [38], stimulating the inducible NO synthase (iNOS)-dependent NO production at transcriptional level, at least partially through activation of the NF-kB-dependent pathways [39]. In mice increased iNOS-derived NO production after RSV infection contributes to both airway inflammatory changes and airway dysfunction, while inhibition of NO synthesis significantly reduces pulmonary inflammation and airway hyperresponsiveness [40]. Consistently, treatment of RSV-infected mice with an antioxidant (butylated hydroxyanisole) induced a significant reduction of RSV-induced pulmonary cytokine production with inhibition of lung recruitment of inflammatory cells, especially neutrophils, which are the major cell type responsible for oxidative burst in response to infectious stimuli [41]. Production of proinflammatory and prooxidant cytokines in response to RSV infection is induced by recognition by toll-like receptors (TLR) of viral double-stranded RNA, produced during viral replication. The downstream signaling pathway from TLR leads to activation of interferon (IFN) regulatory factor-3 and/or NF-kB and subsequent expression of numerous pro-inflammatory factors. Interestingly, it has been shown that melatonin decreases the TLR- mediated downstream gene expression in RSV-infected macrophages and the subsequent NF-kB-dependent gene expression, such as those encoding for TNF-α and iNOS [42]. These results suggest that in RSV infection melatonin may at least in part prevent the injury to the airway structure through the inhibition of the oxidative stress and of the production of proinflammatory cytokine and therefore be a useful therapeutic agent in RSV-induced pulmonary disease. Recent evidence suggests that respiratory disorders induced by many other human viral pathogens may result from exuberant generation of reactive oxygen species by inflammatory cells in response to infection [43]. Finally melatonin has been reported to be effective in human studies performed on in infants with disorder characterized by excessive inflammatory reaction and oxidative damage, overwhelming the physiologic anti-inflammatory/antioxidant signaling processes. Indeed, positive effects of melatonin treatment have been reported in: a) newborns with sepsis, with reduction of serum levels of lipid peroxidation products and with increased survival [44]; b) in preterm infants with respiratory distress syndrome, reducing early serum indicators of chronic lung disease, i.e. plasma concentrations of IL-6, IL-8, TNF-α and of nitrite/nitrate [45]; c) in newborns with respiratory distress syndrome, reducing the levels of proinflammatory cytokines IL-6, IL-8, TNF-α in tracheobronchial aspirate and improving the clinical outcome [46].

## Conclusions

Modulation of the inflammatory response and of the reactive oxygen species production and of the related oxidative stress therefore represents a potential novel pharmacological approach to ameliorate the host reactions against viral infections and their long-term consequences [47,48]. One possible weakness of melatonin is its short half-life and the relatively low levels in serum during day hours [11,14]. Administration of melatonin to humans at pharmacological concentrations is essentially non-toxic, also in the neonatal period [49] and the results obtained *in vitro* and in experimental animals support a possible beneficial immunoregulatory and anti-oxidant role of this molecule in viral infection and address its possible therapeutic potential in human virus-induced diseases.

## Abbreviations

NK cells: Natural killer cells; IL-1: Interleukin −1; TNF-α: Tumor necrosis factor- α; NF-kB: Nuclear factor-kappa B; CNS: Central nervous system; RSV: Respiratory syncytial virus; NO: Nitric oxide; •OH: Hydroxyl radical; MDA: Malondialdehyde; GSH: Glutathione; SOD: Superoxide dismutases; iNOS: Inducible nitric oxide synthase; TLR: Toll-like receptor.

## Competing interests

The authors declare that they have no competing interests.

## Authors’ contributions

All the authors have made substantial contributions to conception and design of the study, to the analysis, interpretation and acquisition of the data. All the authors have been involved in drafting the manuscript and revising it critically for important intellectual content. Both authors read and approved the final manuscript.
